# Supplementary material for: Patterns of Intron Gain and Loss in Fungi
Source: PLoS Biol. 2004 Nov 30;2(12):e422. doi: 10.1371/journal.pbio.0020422 (PMC532390; doi:10.1371/journal.pbio.0020422)
Supplement: Table S1 — Also available at http://genes.mit.edu/NielsenEtAl/. (4.3 MB ZIP). [file pbio.0020422.st001.zip › NielsenEtAl/html/54.html]

AN3468.1.NCU02437.1.MG03577.1.FG11627.1


```
 CLUSTAL W (1.82) Multiple Sequence Alignments - Introns Inserted


Sequence 1: NCU02437.1	134 aa
Sequence 2: MG03577.1	136 aa
Sequence 3: FG11627.1	134 aa
Sequence 4: AN3468.1	132 aa
Alignment Length: 136 aa
Number Identitical Residues: 126 aa
Alignment Score (without introns) 4496


MG03577.1 	MTGGGKSGGKASGSKNAQS2RSSKAGLAFPVGRVHRLLRKGNYAQRVGAG~APVYLAAVL
NCU02437.1	MTGGGKSGGKASGSKNAQS2RSSKAGLAFPVGRVHRLLRKGNYAQRVGAG~APVYLAAVL
FG11627.1 	MTGGGKSGGKASGSKNAQS2RSSKAGLAFPVGRVHRLLRKGNYAQRVGAG~APVYLAAVL
AN3468.1  	MTGG-KSGGKASGSKNAQS2RSSKAGLAFPVGRVHRLLRKGNYAQRVGAG1APVYLAAVL
          	**** ************** ****************************** *********

MG03577.1 	EYLAAEILELAGNAARDNKKTRIIPRHLQLAIRNDEELNKLLGHVTIAQGGVLPNIHQN1
NCU02437.1	EYLAAEILELAGNAARDNKKTRIIPRHLQLAIRNDEELNKLLGHVTIAQGGVLPNIHQN1
FG11627.1 	EYLAAEILELAGNAARDNKKTRIIPRHLQLAIRNDEELNKLLGHVTIAQGGVLPNIHQN1
AN3468.1  	EYLAAEILELAGNAARDNKKTRIIPRHLQLAIRNDEELNKLLGHVTIAQGGVLPNIHQN1
          	*********************************************************** 

MG03577.1 	LLPKKTGKTAGGKNASQEM
NCU02437.1	LLPKKTGKT--GKNASQEL
FG11627.1 	LLPKKTGKT--GKTSSMEL
AN3468.1  	LLPKKTPKA--GKGS-QEL
          	****** *:  ** :  *:
```
